# GPCRdb: an information system for G protein-coupled receptors

**DOI:** 10.1093/nar/gkv1178

**Published:** 2015-11-17

**Authors:** Vignir Isberg, Stefan Mordalski, Christian Munk, Krzysztof Rataj, Kasper Harpsøe, Alexander S. Hauser, Bas Vroling, Andrzej J. Bojarski, Gert Vriend, David E. Gloriam

**Affiliations:** 1 Department of Drug Design and Pharmacology, University of Copenhagen, Jagtvej 162, DK-2100 Copenhagen, Denmark; 2 Department of Medicinal Chemistry, Institute of Pharmacology, Polish Academy of Sciences, Smetna 12, 31-343 Krakow, Poland; 3 Bio-Prodict B.V., Nieuwe Markstraat 54E, 6511 AA, Nijmegen, The Netherlands; 4 CMBI, NCMLS, Radboud University Nijmegen Medical Centre, Geert Grooteplein Zuid 26-28, 6525 GA, Nijmegen, The Netherlands

## Abstract

Recent developments in G protein-coupled receptor (GPCR) structural biology and pharmacology have greatly enhanced our knowledge of receptor structure-function relations, and have helped improve the scientific foundation for drug design studies. The GPCR database, GPCRdb, serves a dual role in disseminating and enabling new scientific developments by providing reference data, analysis tools and interactive diagrams. This paper highlights new features in the fifth major GPCRdb release: (i) GPCR crystal structure browsing, superposition and display of ligand interactions; (ii) direct deposition by users of point mutations and their effects on ligand binding; (iii) refined snake and helix box residue diagram looks; and (iii) phylogenetic trees with receptor classification colour schemes. Under the hood, the entire GPCRdb front- and back-ends have been re-coded within one infrastructure, ensuring a smooth browsing experience and development. GPCRdb is available at http://www.gpcrdb.org/ and it's open source code at https://bitbucket.org/gpcr/protwis.

## INTRODUCTION

G protein-coupled receptors (GPCRs) constitute the largest family of membrane proteins with approximately 800 members in human (1). Roughly half are sensory mediating olfaction, vision, taste and pheromone recognition (2). Of the non-sensory receptors, two-thirds regulate a plethora of physiological processes ranging nervous and endocrine systems, whereas the remaining (∼120 receptors) are still orphan receptors with unknown endogenous ligand and/or function (IUPHAR/BPS Guide to PHARMACOLOGY database, GtoPdb). GPCRs have been systematized based on sequence homology into the classes A–F (3) or the GRAFS families (acronym based on the prototypical members) (4). These are further subdivided into receptor families based on their endogenous ligands that span ions, neurotransmitters, lipids, carbohydrates, nucleotides, amino acids, peptides and proteins (5).

GPCRs constitute the targets of more than a quarter of all FDA approved drugs (6), but the majority, including 58% of the Class A receptors, are still unexploited in therapies/trials (7). A considerate number of receptors, both orphan and liganded, are still actively being pursued to characterize receptor functions/networks and to establish target disease validation. Thanks to a deeper insight into pharmacological mechanisms; such as polypharmacology, allosteric modulation of the physiological activity and ligand-dependent biased signalling through specific signalling protein profiles; there are now alternative concepts to attempt a favourable therapeutic response (8). Furthermore, the recent flurry of X-ray activities has made available a large number of templates for GPCR structure-based drug design.

Crystal structures are now available for all of the human GPCR classes. These have revealed mechanisms of orthosteric and allosteric (9) ligand binding, conformational changes upon receptor activation (10–12), and the modes of binding of G protein (13,14) and β-arrestin (15) signal mediators. The transmembrane domain of GPCRs consists of seven helices forming the signal transduction machinery. This domain also comprises the orthosteric binding site of Class A and B1 (Secretin family) receptor ligands, whereas it acts as an allosteric site for modulation of Class B2 (Adhesion family), C and F GPCRs (16) that bind their natural ligands within their extracellular N-terminus. On the intracellular side, the helical bundle, loops and C-terminus bind G proteins and/or β-arrestin (8).

The GPCR database, GPCRdb was started in 1993 by Gert Vriend, Ad IJzerman, Robert Bywater and Friedrich Rippmann. Over two decades, GPCRdb evolved to be a comprehensive information system, storing and analysing GPCR data (17–20). In 2013, the stewardship of GPCRdb was transferred to the David Gloriam group at the University of Copenhagen, backed up by an international team from the EU COST Action ‘GLISTEN’ (21). The latest release of GPCRdb offers access to a variety of updated and new experimentally derived data: (i) All GPCR crystal structures in the PDB, as well as 309 ligand fragments from crystal structures that may be used to build new pharmacophores or even *de novo* ligands; (ii) The largest collection of GPCR single-point mutations, which may now be deposited directly by users and include observed effects on ligand activities; and (iii) Reference sequence alignments that build on structural annotation ensuring that the residues aligned in sequence are those that reside at the corresponding structure position. Furthermore, GPCRdb features a powerful yet simple-to-use suite of analysis tools and visualization diagrams. Below, these are all described as provided in the menu system for: GPCRdb, Receptors, Sequences, Structures, Mutations, Sites, Residue numbers and Links.

## GPCRDB INFRASTRUCTURE IMPROVEMENTS

### Consolidation under Python and Django, and open source release

The GPCRdb infrastructure has been re-coded, and consolidated into an integrated system written in Python (v3.4), using the Django Framework (v1.8) and the PostgreSQL database management system (v9.3). This allows for a responsive plug-in-independent web browser experience, and for the web interface to access the same core functionality as the back-end, which is advantageous for day-to-day maintenance, as well as for new developments. The complete GPCRdb source code is freely available at https://bitbucket.org/gpcr/protwis under the Apache 2.0 license.

### Virtual machine

GPCRdb provides a virtual machine for running a local version of the resource. This is ideal for developers interested in building on top of GPCRdb functionality, or contributing to the project. The virtual machine can also be used to maintain a local version, e.g. to combine the GPCRdb data with proprietary data or where IT policies prohibits the use of external databases. Instructions for setting up the virtual machine on Linux, Mac and Windows are freely available at https://bitbucket.org/gpcr/protwis_vagrant.

### Web services

GPCRdb now offers REST web services to enable programmatic access to receptor, family, mutation and structure data, as well as sequence alignments and diagrams. The services allow access to data through the Hypertext Transfer Protocol (HTTP) and serve a response in Javascript Object Notation (JSON) format. The services were built using Django REST Framework (v3.1). Examples of how to access the data using Python are provided at http://docs.gpcrdb.org/web_services.html.

## RECEPTORS

The Receptors menu gives direct access to all data for one receptor or receptor family, in contrast to other menu items that represent one data type across multiple receptors. GPCRdb contains all human GPCRs, except the olfactory receptors and all species orthologues in TrEMBL (22). The receptor nomenclature follows that of NC-IUPHAR (23), also listing the gene and alternative protein names from Uniprot. Receptors are hierarchically organized by class, endogenous ligand type, receptor family and subtype; e.g. Class A—peptide receptors—Angiotensin receptors—AT_1_ receptor. The receptor families can be browsed and analysed in the same way as the individual receptors throughout the database. It is, for example, possible to depict their common mutation data, and to use their consensus sequences to generate sequence alignments and phylogenetic trees.

### Residue diagrams – snake and helix box diagrams

**Figure 1.**
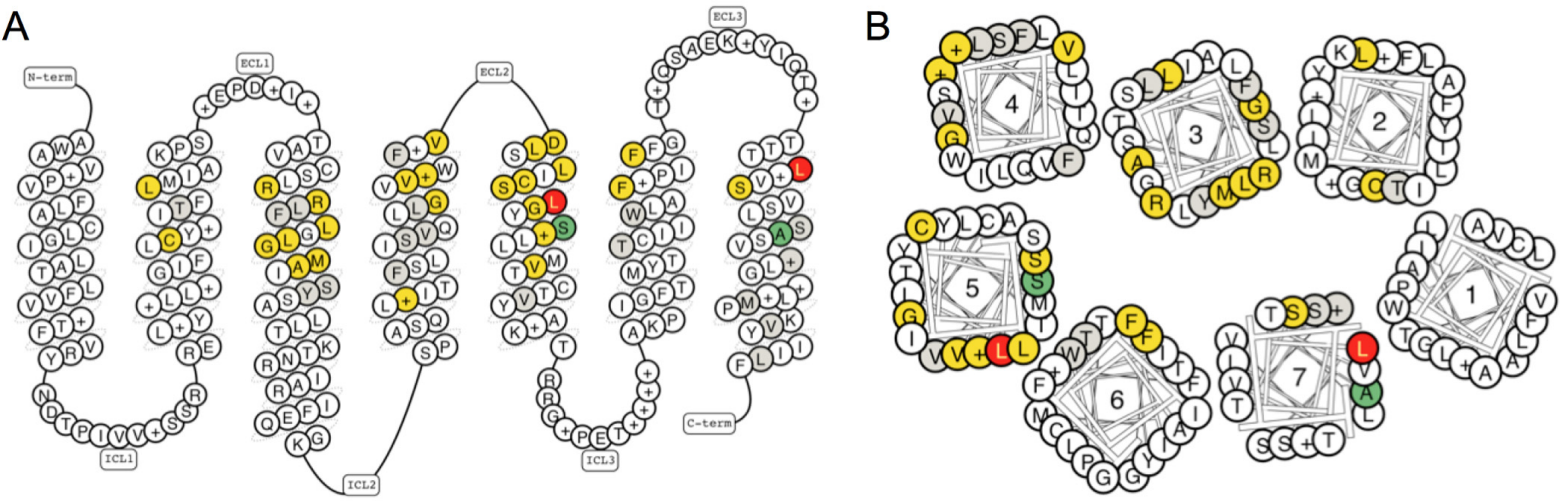
(**A**) Snake and (**B**) helix box diagrams show the receptor residue topologies as seen from the side and top, respectively. In the latest installment of the GPCRdb, the diagrams are used to visualize physico-chemical properties, mutation (fold) effects on ligand binding, and ligand interactions from crystal structure complexes. This example depicts the consensus sequence of the human metabotropic glutamate receptors with colour coding of mutation effects on ligand binding. Colour scheme; Increased binding/potency: >5-fold (light green), >10-fold (dark green); Reduced binding/potency: >5-fold (light red), >10-fold (dark red); <5-fold/no effect (yellow); and unknown effect (gray).

The snake and helix box diagrams visualize receptor residue topologies as seen from the side and above of the cell membrane, respectively (Figure 1A–B). Snake-like diagrams now include full termini, loops, as well as helix 8. The helix box diagrams are designed to best represent the 3D receptor structure, for example helices have four sides because a helical turn contains approximately four residues. Furthermore, helix bulges or constrictions (observed in structures) can offset structural and sequence alignments (24), but are corrected for by single amino acid insertion and removal, respectively. Residue numbers can be displayed by hovering the cursor. Amino acids can be coloured to illustrate residue physico-chemical properties, mutation data presence or mutation effects on ligand binding or potencies, where available. The diagrams can be downloaded as a picture file or in scalable vector graphics format for further editing.

## STRUCTURES

### Structure data

**Figure 2.**
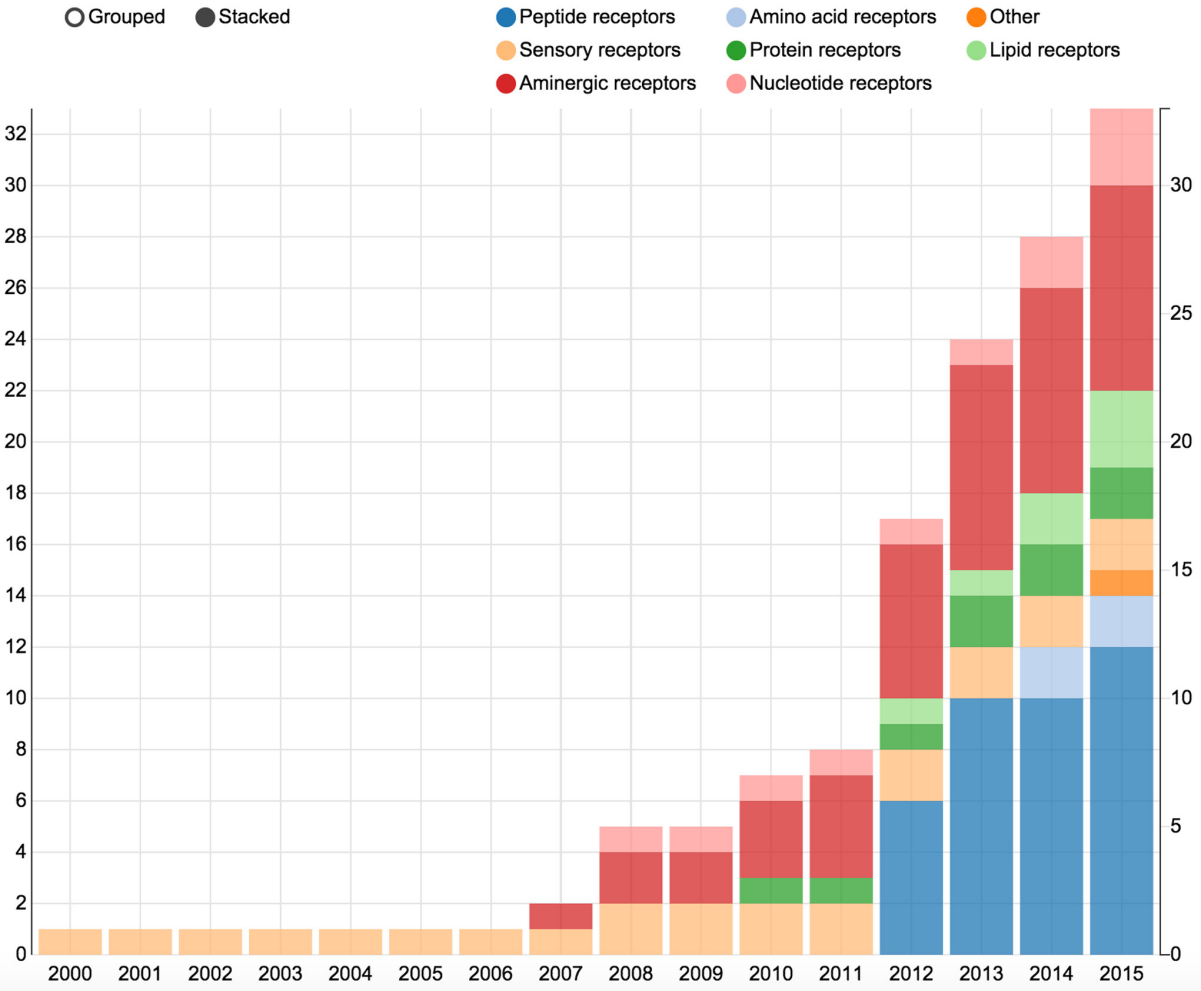
The bar diagrams in the Structure statistics page plot the number of unique or total crystallized GPCRs in the protein data bank (PDB) (25) by year and the colours indicate the type of ligand.

**Figure 3.**
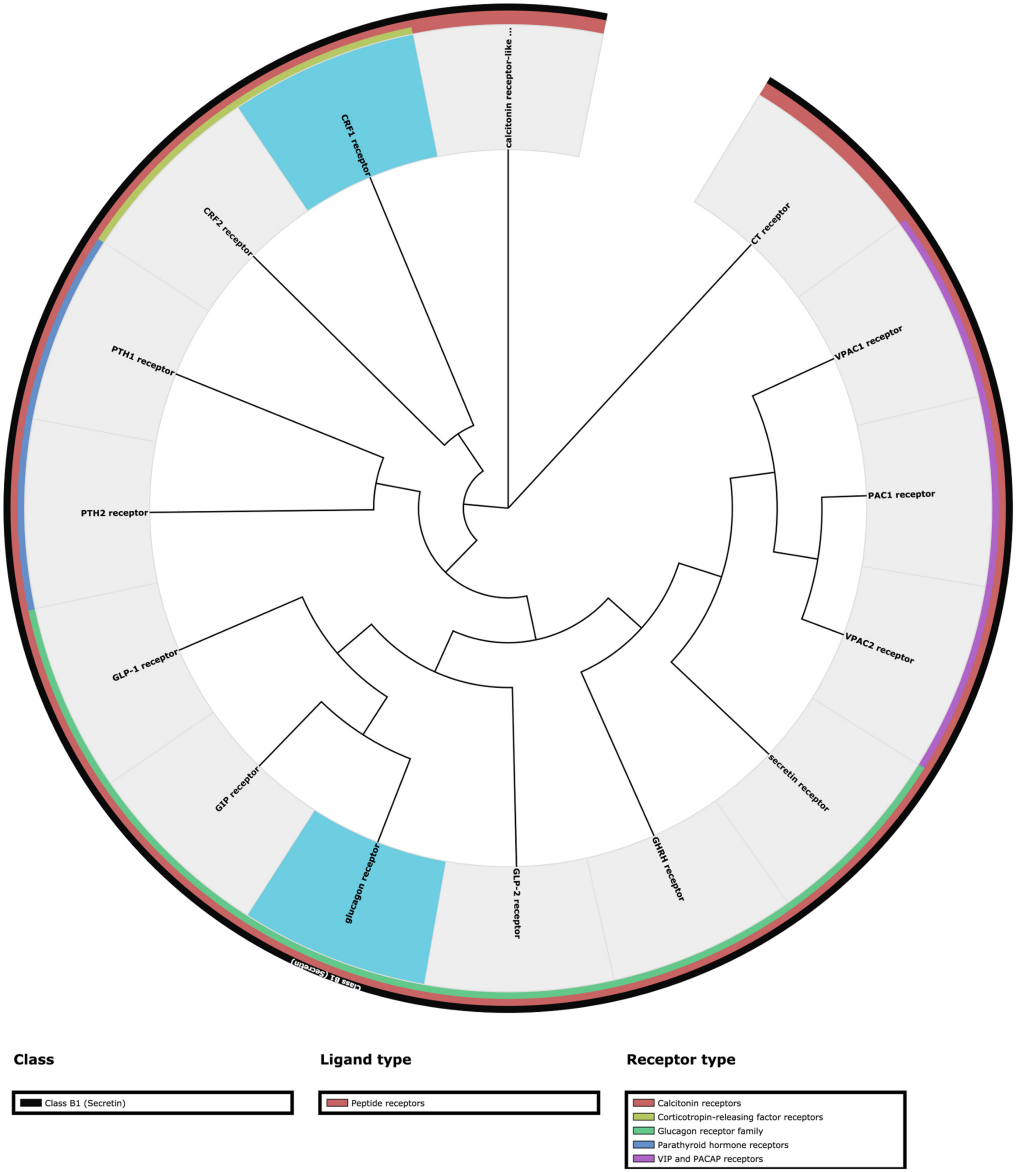
This figure shows the human Class B1 (Secretin family) GPCRs. Custom Phylogenetic trees can swiftly be calculated under the Sequences tools menu. The interpretation of the trees is guided by coloured bars next to the receptor names that list the GPCR class, ligand type and receptor family, respectively. Receptors that have been crystallized can be highlighted with a blue background, and other background colours can be manually assigned to produce custom illustrations. Pre-generated phylogenetic trees available in the Structure statistics page show the structural coverage within each GPCR Class. For Class A, the consensus sequences are used for receptor families without a structure, whereas all receptor subtypes are included for the other Classes.

Hitherto, 132 GPCR structures have been reported for 30 unique receptor subtypes. Although wonderful to have, all these data pose challenges in keeping track of what is available, and what is the most relevant receptor (ligand complex) for a given study. The Structure statistics page features bar diagrams that depict the number of unique or total crystallized GPCRs in the protein data bank (PDB) (25) split by ligand type or year (Figure 2), and phylogenetic trees (Figure 3) that show the coverage within each GPCR Class. The Structure browser allows for the selection of GPCR structures based on human annotations of receptor classes, crystallized and endogenous ligands, auxiliary proteins and structure properties, such as resolution.

### Structure tools

The structural tools are available both as separate tools and via the Structure browser. They process user-uploaded (model) and browsed (crystal) structures, respectively. The PDB file residue numbering tool assigns generic numbers to PDB files (see below). The Superposition tool allows users to superpose GPCR structures based on the overall or any substructure, e.g. ligand binding residues, and download in PDB format. The Download tool also supports overall or custom substructures. Finally, the Template selection tool for homology modelling extends the crystal structure browser with sequence similarities for a user-defined target.

## SEQUENCES

### Structure-based sequence alignments

GPCRdb features alignments of all human GPCR classes A–C and F (D and E are non-human), covering all non-olfactory human receptors (398) and species orthologues in Swiss-Prot and TrEMBL (>18 000). Traditionally, sequence alignments have been based solely on conserved sequence motifs, and where available, supporting mutagenesis data. This is problematic as some transmembrane helices and/or receptors lack such conserved motifs, especially between the GPCR classes. Furthermore, the seven transmembrane helices frequently contain irregularities, i.e. bulges or constrictions, introducing insertions and gaps, respectively, as compared to a normal helix followed by an alignment offset (24). GPCRdb has capitalized on this wealth of structure information and replaced the traditional sequence-based alignments with structure-based alignments that are better for many applications including inference of mutation effects, comparison of binding sites and homology modelling (26). Such alignments ensure residue correspondences by aligning the same residues in sequence as aligned in superposition of GPCR crystal structures, as described hereunder.

First, a manual annotation takes place by superposition of representative (inactive) structures for crystallized GPCRs. This annotation defines (i) the location of the generic numbering reference position (X.50) within the receptor protein sequence, (ii) the ends of structural conservation for compared segments; transmembrane helices and helix 8; and (iii) any transmembrane helix bulges or constrictions. On this basis, a reference crystal structure-based sequence alignment is built in which residues are only aligned in sequence if they reside in the equivalent structural position. Subsequently, non-crystallized receptors are appended to the alignment by assigning them the same structural information as their most homologous crystal structure – this is the same principle as in homology modelling. Finally, a correction is made in cases where a specific sequence motif has been found to induce a certain bulge or constriction configuration. Thus, retrieval of any GPCRdb alignment represents the look-up of a subset from this overall all-receptor alignment. Below each alignment, three forms of residue conservation statistics are presented: (i) a consensus sequence, (ii) the prevalence of the 20 amino acids, and (iii) shared properties, such as aromaticity, charge or hydrogen bonding ability.

### Sequence tools

Sequence similarity is often used to deduct homology and to infer shared physiological functions. GPCRdb includes BLAST similarity search (27) to quickly find the most similar sequences for a user-provided query. The GPCRdb similarity search also makes query-to-database searches, but uses the reference structure-based alignments for both queries and hit sequences, and is able to restrict the search to specific segments – custom sets of transmembrane helices or residues (by generic numbers). Hits are aligned in order of sequence identity, similarity or alignment score; and can be downloaded as either an alignment file (fasta) or a spreadsheet (csv).

All-to-all receptor comparisons of sequence identities and similarities can be displayed in a similarity matrix. Phylogenetic trees (28) can be generated with up to 100 bootstraps based on any GPCR 7TM subsequence. They can be displayed in circular and ladder representations, and downloaded as figures or in Newick format for use in tree viewing software. The interpretation of the trees is guided by coloured bars next to the receptor names that list the GPCR class, ligand type and receptor family, respectively (Figure 3). Furthermore, receptors that have been crystallized can be highlighted with a blue background, and other background colours can be manually assigned to produce custom illustrations.

## MUTATIONS

Literature contains vast amounts of mutagenesis studies pinpointing receptor residues involved in ligand binding and efficacy. GPCRdb holds a large collection of manually annotated such mutations, currently over 12 000 mutations for 192 receptors (21,29). Previously, only the minimal information has been stored: the receptor, residue number, wild type and mutation amino acids and a reference publication. In the last year, the annotation was extended with the effect on ligand affinity or potency, and receptor surface expression or basal activity. We added 1536 new mutations, including all published Class C GPCR mutations residing in the transmembrane domain. Users can now themselves submit mutations to generate illustrations for publication (below) or to increase the dissemination of already published studies. To ensure that data from different sources are uniform, the deposition uses an excel file in which data are described using controlled vocabularies and identifiers from public databases.

**Figure 4.**
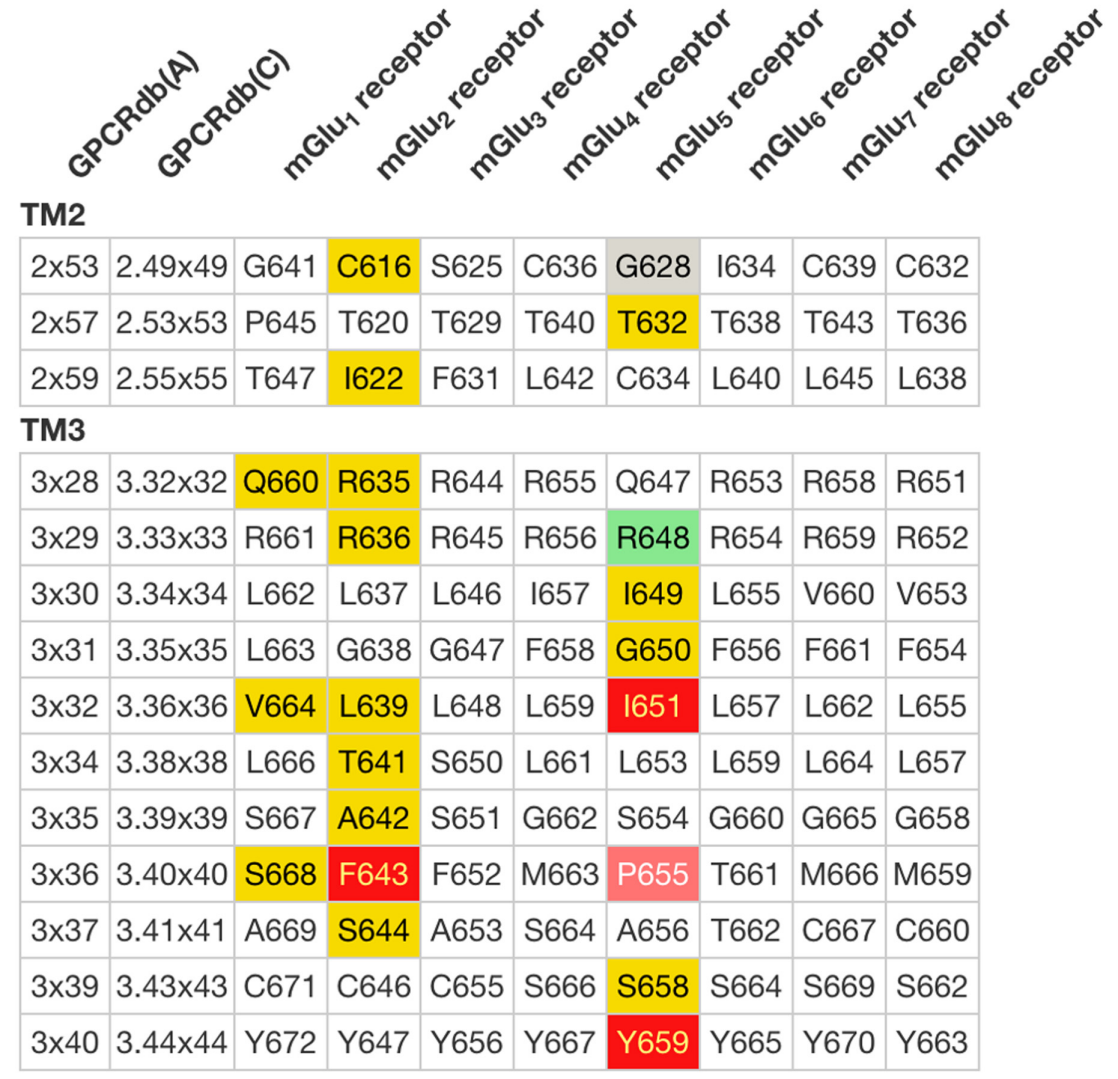
Residue Tables give a side-by-side comparison of subtype residues lined up by their common generic residue number. Like the Residue Diagrams (Figure 1), they can be colour coded to visualize physico-chemical properties, mutation (fold) effects on ligand binding and ligand interactions from crystal structure complexes. This Residue Table depicts the human metabotropic glutamate receptor mutations in the transmembrane helices 1–2 coloured according to their effects on ligand binding (see Figure 1).

The Mutation browser allows for browsing and download of mutations for specific receptors and their subdomains. The Residue diagrams, snake-plots and helix box diagrams (Figure 1), can be custom-coloured or coloured automatically to highlight the effects of mutations on ligand binding or potency. Residue tables show the same colour coding as the diagrams and allow for the comparison of mutations across receptor families and subtypes (Figure 4).

## SITES

### Ligand interactions

**Figure 5.**
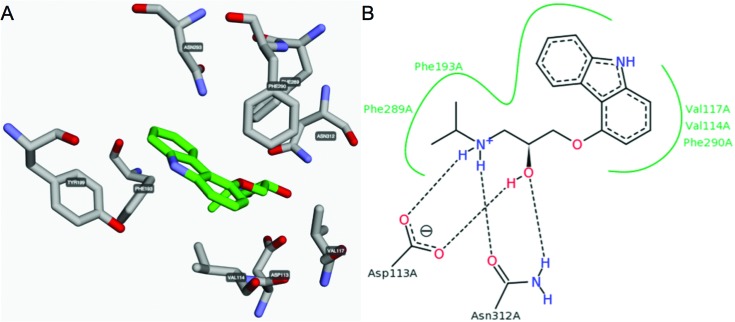
Receptor–ligand interactions from structure complexes (pdb file upload) can be visualized either in (**A**) a rotatable 3D structure viewer or (**B**) a schematic 2D interaction diagram.

Ligand binding sites have been annotated for all GPCR-ligand complexes in the PDB, and can be automatically generated for a user-uploaded pdb file, e.g. from ligand docking in a receptor model. The predefined binding sites can be accessed on per structure basis, and currently cover 1201 ligand interactions from 107 complexes of 29 receptors with 65 ligands. The receptor–ligand interactions can be visualized in a rotatable 3D structure viewer and schematic 2D interaction diagram implemented with 3dmol.js (http://3dmol.csb.pitt.edu) and PoseView (30), respectively (Figure 5). Furthermore, as for the mutations, ligand interactions can also be visualized in Residue diagrams, helix box and snake diagrams (Figure 1), and compared in Residue tables (Figure 4) across receptor families and subtypes.

### Site tools

The Site search tool matches a sequence/structure site, such as a ligand binding site or structural motif, against the GPCRdb reference alignments to retrieve a set of matching receptor (off-) target profiles. This is relevant to rationalize observed polypharmacology, select receptor panels for off-target screening, or ligand inference from old to new targets. The site search tools differs from the above sequence similarity tools in that it discriminates receptors into matches and non-matches, and that each residue position is assigned a specific set of allowed amino acids, specifically only those that have the desired functional property such as hydrophobicity, hydrogen bond donor capability and size. This GPCRdb update has made this tool significantly more accessible by simplifying the manual site definition, and adding an automatic definition of ligand binding sites by upload of a receptor–ligand pdb file.

The Pharmacophore generation tool for GPCR binding sites (31) is based on the inference of crystal structure fragments between homologous receptors, and does therefore not require known ligands or receptor structures for the target of interest. A pharmacophore fragment is the pair of one ligand moiety interacting with one receptor residue. The annotated fragment library has in total 309 such fragments that cover 29 residue positions within the generic transmembrane binding pocket (32). Users can automatically select a crystal structure template, match and superpose fragments (all or representative) to download a single zip file containing the receptor structure and all superposed matching fragments in pdb format.

## RESIDUE NUMBERS

**Figure 6.**
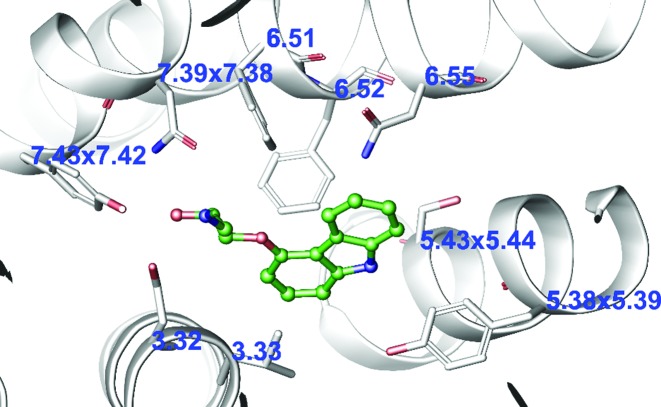
Display of generic residue numbers inserted into a pdb file and visualized with the Maestro labelling plugin. This example shows the sequence-based Ballesteros–Weinstein numbers, and where these are offset by helix distortions, the structure-based GPCRdb residue numbers (24) within the crystal structure of β_2_-adrenergic receptor (pdb: 2RH1).

A generic residue number gives an index of a transmembrane helix residue position within a multiple sequence or structure alignment. GPCRdb provides all the established sequence-based residue numbering schemes for the Classes A: Ballesteros and Weinstein (33), B: Wootten *et al*. (34), C: Pin *et al*. (35) and F: Wang *et al*. (36). Recently, GPCRdb also introduced a structure-based GPCRdb numbering, which is the same as the above but corrects for structural distortions, helical bulges and constrictions, to ensure that the residues aligned in sequence are those that align in structure (24). In GPCRdb, residue numbers can be retrieved directly in the Structure-based sequence alignments or within Residue tables (Figure 4). The PDB file residue numbering tool assigns numbers to any user-provided structure (Figure 6).

## LINKS

GPCRdb is cross-referenced to the IUPHAR/BPS GuideToPharmacology (GToPdb) (5), UniProt (22) and BitterDb (37) databases. GPCRdb has adopted the official NC-IUPHAR receptor names, and have made available to NC-IUPHAR the receptor residue diagrams and mutations for direct access in GToPdb. GPCRdb and GToPdb contain complementary receptor structure/sequence, and ligand/pharmacological data, respectively, and the release of harmonized web services will allow users to combine data from the two resources. Furthermore, GPCRdb contains links to several specialized GPCR servers for homology modelling: GPCRM (38), GoMoDo (39) and SSFE (40); and molecular dynamics: GPCR Mod-Sim (41). Our developers continuously exchange information, e.g. at the biannual GPCRdb satellites at the GLISTEN EU Cost meetings, and the GPCRdb web services may be used by partners to retrieve reference data, such as the structure-based sequence alignments. For information on how to link to GPCRdb, visit http://docs.gpcrdb.org/linking.html.

## CONCLUSIONS AND FUTURE PERSPECTIVES

In conclusion, the fifth release of GPCRdb offers experimental and derived data, visualization diagrams and analysis tools for the wider GPCR community. The move to one integrated system makes the user-interface more responsive and facilitates the development. The manual data annotation encompasses on GPCR crystal structures, sequence alignments, ligand fragments and single-point mutations. Powerful yet simple tools facilitate browsing, retrieval, querying, and inference of receptor overall and subdomain information. Several new or improved interactive visualization options allow for online analysis and diagram download. Collectively, this is in line with and could contribute to the ongoing advances in receptor structure and function, as well as structure-based drug design.

GPCRdb is the first to offer structure-based GPCR sequence alignments and generic residue numbers (24). As can be seen from the three community-wide ‘GPCR Dock’ assessments (42–44), there is a large interest and expertise in GPCR homology modelling, and the increasing number of structural templates have led to higher precision. GPCRdb is currently working towards providing built-in models of all human receptors based on technologies that gave the best receptor RMSD for the serotonin 5-HT_1B_ receptor (44). These involve the use of alternative templates for specific substructures and GPCR position-specific rotamer libraries extracted from crystal structures.

GPCRdb allows for complementary data types to be assigned to receptor residue positions and visualized within uniform residue diagrams and tables. This allows users to integrate evolutionary conservation, pharmacological effects of single-point mutations and ligand interactions from crystal structure complexes (45). Users have the option to submit new mutation data to facilitate comparison with the data in GPCRdb. Furthermore, GPCRdb is in the works of a combined visualization of the above data types within one simultaneous diagram.

GPCRdb features a multi-residue site search that retrieves the profile of receptor targets that share the given site, and by inference, also it's associated function. This can now be readily applied to analyses of structural motifs stabilizing an (in)active receptor conformation, or ligand binding sites to assess ligand selectivity or polypharmacology. It is expected that many more GPCR crystal structure complexes will become available that provide further insights into activation mechanisms, and the molecular sites of G protein (13,14) and β–arrestin (15) binding, and receptor dimerisation (46–49). The GPCRdb is ready to absorb these data and aid the GPCR research community with their dissemination.
