# GPCRdb: an information system for G protein-coupled receptors

**DOI:** 10.1093/nar/gkw1218

**Published:** 2016-12-06

**Authors:** Vignir Isberg, Stefan Mordalski, Christian Munk, Krzysztof Rataj, Kasper Harpsøe, Alexander S. Hauser, Bas Vroling, Andrzej J. Bojarski, Gert Vriend, David E. Gloriam

**Affiliations:** 1 Department of Drug Design and Pharmacology, University of Copenhagen, Jagtvej 162, DK-2100 Copenhagen, Denmark; 2 Department of Medicinal Chemistry, Institute of Pharmacology, Polish Academy of Sciences, Smetna 12, 31-343 Krakow, Poland; 3 Bio-Prodict B.V., Nieuwe Markstraat 54E, 6511 AA, Nijmegen, The Netherlands; 4 CMBI, NCMLS, Radboud University Nijmegen Medical Centre, Geert Grooteplein Zuid 26-28, 6525 GA, Nijmegen, The Netherlands

*Nucl. Acids Res*. (2016) 44 (Database issue): D356–D364. doi: 10.1093/nar/gkv1178

The Authors would like to add a funding acknowledgement to their paper.

“The COST Actional CM1207 ‘GLISTEN’ has generously hosted thebiannual GPCRdb meetings and supported multiple research visits.”

should be replaced by

“This article is based upon work from Action GLISTEN (www.glisten-gpcr.eu), supported by COST (European Cooperation in Science and Technology, www.cost.eu), which is supported by the EU Framework Programme Horizon 2020. Gáspár Pándy–Szekeres helped with structural data annotation.”

This has been corrected online.

